# Machine-Aided Bridge Deck Crack Condition State Assessment Using Artificial Intelligence

**DOI:** 10.3390/s23094192

**Published:** 2023-04-22

**Authors:** Xin Zhang, Benjamin E. Wogen, Xiaoyu Liu, Lissette Iturburu, Manuel Salmeron, Shirley J. Dyke, Randall Poston, Julio A. Ramirez

**Affiliations:** 1 Lyles School of Civil Engineering, Purdue University, West Lafayette, IN 47907, USA; zhan3794@purdue.edu (X.Z.); bwogen@purdue.edu (B.E.W.); liturbur@purdue.edu (L.I.); salmeron@purdue.edu (M.S.);; 2 School of Mechanical Engineering, Purdue University, West Lafayette, IN 47907, USA; liu1787@purdue.edu; 3 Pivot Engineers, Austin, TX 78746, USA; poston@pivotengineers.com; 4 Department of Civil and Environmental Engineering, University of Texas at San Antonio, San Antonio, TX 78249, USA

**Keywords:** machine-aided bridge inspection, deep learning, image classification, semantic segmentation, risk management

## Abstract

The Federal Highway Administration (FHWA) mandates biannual bridge inspections to assess the condition of all bridges in the United States. These inspections are recorded in the National Bridge Inventory (NBI) and the respective state’s databases to manage, study, and analyze the data. As FHWA specifications become more complex, inspections require more training and field time. Recently, element-level inspections were added, assigning a condition state to each minor element in the bridge. To address this new requirement, a machine-aided bridge inspection method was developed using artificial intelligence (AI) to assist inspectors. The proposed method focuses on the condition state assessment of cracking in reinforced concrete bridge deck elements. The deep learning-based workflow integrated with image classification and semantic segmentation methods is utilized to extract information from images and evaluate the condition state of cracks according to FHWA specifications. The new workflow uses a deep neural network to extract information required by the bridge inspection manual, enabling the determination of the condition state of cracks in the deck. The results of experimentation demonstrate the effectiveness of this workflow for this application. The method also balances the costs and risks associated with increasing levels of AI involvement, enabling inspectors to better manage their resources. This AI-based method can be implemented by asset owners, such as Departments of Transportation, to better serve communities.

## 1. Introduction

According to the National Bridge Inventory, 42% of the nearly 620,000 bridges in the United States were built more than 50 years ago (NBI) [1]. Furthermore, 12% of all bridges were built more than 80 years ago, exceeding their intended design life of 75 years [2]. To prevent potential safety hazards caused by bridge deterioration, the Federal Highway Administration (FHWA) requires a routine inspection at least every 2 years to evaluate each bridge’s condition [3]. The FHWA and the US Department of Transportation (USDOT) allocate a considerable amount of annual funding towards improving bridge safety by standardizing inspections and evaluations. In May 2022, FHWA and USDOT announced the allocation of an additional $1.14 billion in funding for bridge asset management [4]. Simultaneously, they announced updates to the specifications to be used for future bridge inspections [4]. This announcement stated that, effective after the annual submittal date (the anniversary date of the previous year’s inspection) specified in the FHWA’s Implementation Memo, bridge inspection data should be reported according to *The Specifications for the National Bridge Inventory* (*SNBI 2022*) [5]. This specification will supersede the former *Recording and Coding Guide for the Structure Inventory and Appraisal of the Nation’s Bridge* (*Coding Guide 1995*) [6]. 

A major change in the *SNBI 2022* includes condition assessments for a more detailed set of bridge elements, adopting the bridge element-level inspection system from the *Manual of Bridge Element Inspection* (*MBEI*) [7]. While this new specification has the potential to improve the quality of bridge inspections, it may also increase costs. In the *SNBI 2022*, 11 bridge elements require assessment, compared with the six components in the *Coding Guide 1995*. The *SNBI 2022* also includes the bridge element-level inspection system defined by *MBEI*, which attempts to quantitatively associate element defects with one of four condition states. Their definitions take into consideration the bridge element type, element material/construction type, the relevant damage types, and damage measurements. Consequently, this new system will provide more data to engineers for future deterioration tracking and modeling. However, these updates may significantly increase the workload of bridge inspectors. In addition, it is possible that as bridges continue to age faster than they are rebuilt, inspection requirements may become even more detailed. Thus, applying innovative and efficient techniques to assist this practice will be crucial.

For many years, researchers have been exploring the application of artificial intelligence for infrastructure inspection. Here we organize the previous research into four levels: (1) image organization; (2) damage detection; (3) information extraction; and (4) damage quantification. The first level of this application is the rapid organization of inspection data for future retrieval. Saha et al. utilized a CNN to classify building images based on the function of the buildings, e.g., schools or non-schools [8]. Yeum et al. used AlexNet [9] to classify post-event building images based on content, including the perspective of the image and the building elements [10]. Zhang et al. made use of ResNet 50 to classify bridge images based the perspective and the bridge element shown in the image [11]. Fend et al. developed a GPU-enabled method to classify pavement distress images in real time [12]. The second level of application is detecting damage from inspection images. The computer vision methods of image classification and object detection have proven successful for this task. Haciefendioglu et al. detected and classified common damage in wooden structures [13]. Ale et al. utilized object detection to automatically find pavement cracks from roadway images [14]. Zhang et al. developed a single-stage detector to mark concrete bridge surface damage [15]. An assembled method utilizing PCA-compressed residual frequency response functions and neural network has been developed by Li et al. to identify damage in civil engineering structures [16]. At this level, damage is found and presented to an engineer; however, the engineer generally still needs to interpret or quantify the damage. The third level of application includes generating relevant information for further evaluation, e.g., material information. For example, Yuan et al. was able to automatically classify common building materials through 3D terrestrial laser scan data [17]. Merono et al. utilized image classification techniques to recognize materials and damage on historical buildings [18]. Bunrit et al. improved the representation of CNN-based features by autoencoder for a task of construction material image classification [19]. Rashidi et al. tested different machine learning techniques for detecting construction materials in digital images [20]. The final level of AI application is damage quantification and measurement. In pavement and concrete crack images, one key step at this level is generating a contour of the damage from images. If a contour can be coupled with a reference object to establish scale, the geometric size of the damage can be calculated. Computer vision algorithms and semantic/instance segmentation techniques are usually used at this level and concrete cracks are a common application. Dinh et al. implemented a computer vision-based method for generating the contours of cracks [21]. Similarly, Dung et al. utilized the deep fully convolutional neural network and achieved the objective of autonomous concrete crack detection [22]. Song et al. improved the performance of detecting cracks from images by pre-processing and fusing the crack images with the crack range images [23,24]. Finally, Choi et al. developed SDDNet which can segment cracks in real time [25].

Despite all of this work and the fact that this research has significantly promoted the application of AI for infrastructure inspection, a robust, AI-based framework able to complete the tasks that will soon be required by the *SNBI 2022*, e.g., assigning condition states to damage, does not yet exist. Thus, this work aims to assist human inspectors by rapidly performing element-level condition state assignments for reinforced concrete bridge decks, as required by the new specification. More specifically, bridge deck cracking was selected to demonstrate the proposed method. Existing AI techniques, including image classification [26] and semantic segmentation [27], were used to generate the information needed for condition state assessment. To build confidence in the accuracy of these techniques, a cost-based decision making method was also developed. This method allows engineers to choose the combination of automation and manual inspection for a given bridge inventory that best aligns with their risk tolerance. 

This paper presents a practical process of machine-aided condition state assessment using AI. The first section, known as *Technical Approach*, includes: (1) an introduction to the element-level inspection tasks required by *SNBI 2022*; (2) the design of an AI-based method for the condition state task; (3) the experimental dataset used for validating the proposed method; and (4) the cost and decision making functions. The second section, known as Experimental Validation, includes the detailed process and results of applying this method to an experimental dataset. In the third section, known as *Illustrative Example*, the decision making method is demonstrated with a simulated dataset to describe the decision making process for implementing manual versus automated assessments.

## 2. Technical Approach

### 2.1. Element-Level Inspections

In 1968, the National Bridge Inspection Program was introduced due to the collapse of the Silver Bridge spanning the Ohio River. Since then, several bridge inspection manuals have been introduced and revised [28]. In the 1970s, three manuals—the FHWA *Bridge Inspector Training Manual 70*, the American Association of State Highway Officials (AASHO) *Manual for Maintenance Inspection of Bridges*, and the *Coding Guide 1995* [6]—were introduced to support bridge inspections [28]. In 1995, the FHWA made significant updates to the *Coding Guide 1995* to provide each state DOT with guidelines for compiling inspection reports [29]. This version of *Coding Guide 1995* requires the assessment of the condition of each bridge’s major components using condition rating numbers from 0 to 9. The ratings are required only to indicate the condition of six major components (e.g., the sub-structure, superstructure, deck, etc.), with 9 being a perfect rating and 0 requiring complete bridge replacement. For the remainder of this paper, this condition rating system will be referred to as component-level inspection.

Component-level inspection has drawbacks. Several studies found that it results in ratings with high uncertainty due to the subjective rating definitions [28]. Additionally, this system focuses only on the major bridge components and neglects assessing more specific bridge elements [30,31]. Thus, a new system was developed for more detailed element-level inspections in 2013, outlined in the first edition of the AASHTO *MBEI* and required by the new *SNBI 2022* manual. In this manual, the major bridge components are further divided and categorized into minor elements based on material and functionality. For example, the bridge deck (component) can be categorized as a reinforced concrete deck or a pre-stressed concrete deck (elements). For each element, a table is provided to correlate element defects with one of four condition states (CSs): good (CS1), fair (CS2), poor (CS3), and severe (CS4). These tables provide a more specific and quantitative relationship between the damage and the condition states than past guidelines. 

As described in AASHTO *MBEI*, condition states are typically assigned based on damage type, damage size, and any maintenance actions previously taken. For example, to assess cracks on a reinforced concrete bridge, deck inspectors need to record the crack type (normal or map cracks), crack size (insignificant, moderate-width, or wide), and previous maintenance actions (sealed or non-sealed). CS1 is assigned to insignificant cracks or to moderate-width cracks that have been successfully sealed. CS2 is assigned to moderate-width cracks that remain unsealed or unsealed moderate pattern (map) cracking. CS3 is assigned to wide cracks or heavy pattern (map) cracking that may require more significant repairs. CS4 indicates that the cracking could influence the strength or serviceability of the element or bridge. As defined in the manual, an insignificant crack width is smaller than 0.012 inches, a moderate crack width is larger than 0.012 inches but smaller than 0.05 inches, and a wide crack width is larger than 0.05 inches. Thus, to assess the condition state for the cracking defect using AI-based methods, all of this information must be extracted from images and then compared with the tables defined by *MBEI*.

### 2.2. Design of the AI-Based Workflow

**Figure 1:**
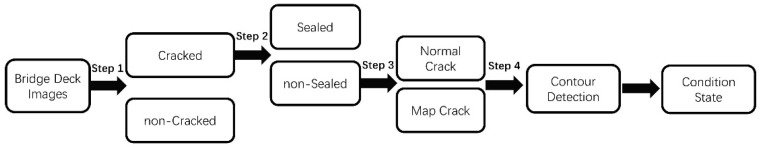
AI-based workflow to determine CSs of bridge deck with cracks.

As described in the previous section, condition states (hereafter, CSs) can be assigned once damage information is extracted from bridge deck images. Computer vision methods can be utilized to automate the extraction of different types of information. Image classification is used to determine image-level (or same level) information, e.g., what the main object is in an image or the pre-set categories to which an image belongs. Semantic segmentation is generally used to determine pixel-level information, e.g., among all of the pixels in an image, the object/category to which each pixel belongs. Thus, semantic segmentation is useful for determining the position and shape of a specific object captured in an image. Based on the complementary features of these two methods, an information extraction workflow was designed specifically for different bridge damage types. In this work, we used reinforced concrete deck cracking to design and demonstrate an AI-based workflow. As summarized in the previous section, the crack type, crack size, and previous maintenance actions must be extracted. The information in CS4 was not considered in this study because the owner will automatically call for manual inspection and this bridge will likely be closed to the public. Furthermore, very few bridge inspection images are available for this stage. Thus, the AI-based method developed here does not apply to decks in this condition. The overall workflow designed for condition assessment is shown in Figure 1.

**Figure 2:**
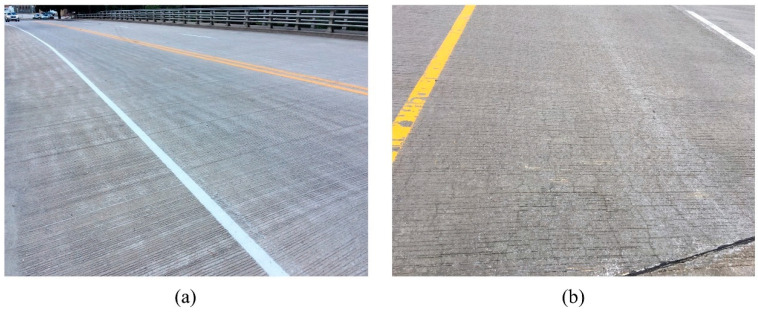
Examples of (**a**) “non-Cracked” images and (**b**) “Cracked” images for Image Classifier I.

**Figure 3:**
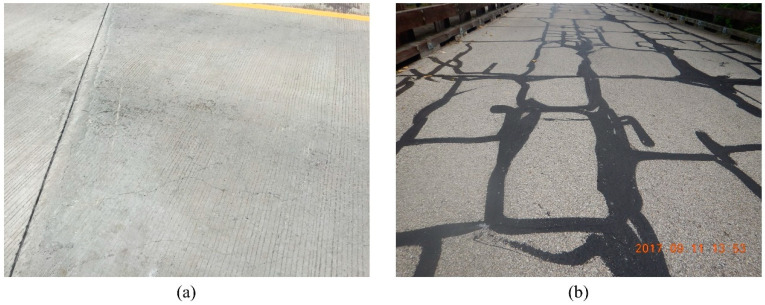
Examples of (**a**) “non-Sealed” images and (**b**) “Sealed” images for Image Classifier II.

**Figure 4:**
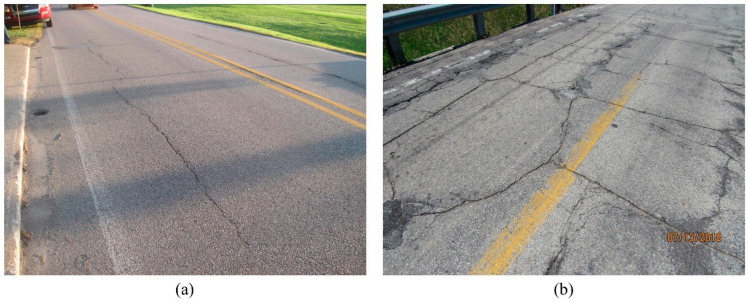
Examples of (**a**) “Normal Crack” images and (**b**) “Map Crack” images for Image Classifier III.

Researchers previously developed an AI-based classification schema which successfully classifies bridge images based on the bridge’s components and elements [11]. Thus, the proposed workflow starts with reinforced concrete bridge deck images, assuming they are already pre-filtered and, thus, only include deck images. In the first step (Step 1), deck images were classified as “Cracked” or “non-Cracked” using image classification. The “Cracked” category includes images that show cracking in the deck, while the “non-Cracked” category includes images with no visible cracking. An image classifier (Image Classifier I) was designed to perform this step. Per description in Section 2.1, the “non-Cracked” category was then automatically assigned to CS1, while the “Cracked” images required further analysis. Thus, in the second step (Step 2) the “Cracked” images were further classified based on whether the crack shown in the images had been sealed (defined as “Sealed” category) or not (defined as “non-Sealed” category). An image classifier (Image Classifier II) was designed to execute this step. The “non-Sealed” images continued to the next step (Step 3) to determine the type of cracks present. The workflow does not determine the crack type for the “Sealed” category because sealing often covers the crack pattern. Consequently, crack patterns could be wrongly identified if they are in the “Sealed” category. In practice, crack patterns are usually recorded before the sealing action and, thus, if a crack is sealed it is reasonable to assume the crack type is already in the database. To determine the crack pattern (Step 3), a third classifier was designed (Image Classifier III) and used to classify images into the “Normal Crack” or “Map Crack” categories. Images in the “Map Crack” category have intersecting cracks that extend below the surface of hardened concrete, forming a network of cracks (resembling a road map) [32]. All other cracks were categorized into the “Normal Crack” category. Image examples of each classifier’s categories, taken from the Bridge Inspection Application System (BIAS) [33] managed by Indiana Department of Transportation (INDOT), are shown in Figure 2, Figure 3 and Figure 4:

In the last step (Step 4) of this workflow, semantic segmentation was used to find the contour of the cracks to estimate their size. Previous research showed how to evaluate the geometric size of an object in an image using this method. Shan et al. developed a method using canny edge detection and Zernike sub-pixel evaluation [34]. A real-time crack geometric size generation method was developed by Li and Zhao using an encoder–decoder network [35]. Other researchers, such as Choi et al. [36] and Liu et al. [37], continued to improve the practice of the AI-based crack measurement method by adding angle adaptability and portability. Since the images in the dataset built for this work were taken through bridge inspectors’ daily work and, thus, were not specifically taken for crack assessment research using computer vision, the authors elected not to re-develop advanced crack size measurement methods. Instead, crack contours were generated using semantic segmentation and then used to quantitatively analyze how the accuracy of these contours influences the decision of assigning a CS. With the information generated through these four steps, the CS of a crack image and, in aggregate, the CS of bridge deck cracking can be assigned to a reinforced concrete bridge deck.

### 2.3. Cost Function to Quantify Loss Due to Misclassification of Condition States

Although researchers have developed many metrics to evaluate the performance of their computer-vision methods, AI methods are not expected to achieve perfect accuracy [38]. For image classification, the measures of accuracy, precision, recall, and f1-scores may be used to evaluate classification results. For semantic segmentation, the measures of pixel accuracy, pixel precision, pixel recall, and intersection over union (IoU) may be used to evaluate performance. However, these conventional AI metrics do not consider different consequences caused by different types of errors. When the final outputs of an AI method are used for a real-world decision, a specific cost table can be formulated to account for these different consequences and evaluate the performance of the AI methods within the context of the problem and workflow.

We note that the overall goal of this workflow is to determine the CS of cracks on a reinforced concrete deck element. Any errors in assigning CSs have varying consequences. In some situations, the misclassification of the CSs may be inconsequential, while in other cases the misclassifications could result in some hazard. For example, if a CS3 (poor) crack is misclassified as CS2 (fair) or CS1 (good), proper maintenance actions for a poor condition crack will not be taken, which may lead to unexpected failures. Considering these situations, a cost function for the final CS prediction, which is composed of estimated real-world costs, is proposed in Table 1.

The parameters in Table 1 reflect conceptual costs for the misevaluation of CSs. The parameters are defined as follows:

$C_{MISij}$—This parameter indicates the misclassification of the CS of the crack on the reinforced concrete bridge deck from CS *i* to CS *j* while *i, j* = 1, 2, 3. When *i* is equal to *j*, this parameter should be 0 because this situation reflects the fact that the classification of the CS is correct (no cost).

$C_{modify}$—This parameter refers to the database modification “fee” when a misclassification of the CS occurs.

The specific values assigned to these parameters should be determined at the local level, considering an inspection region’s economy, natural and traffic hazards, maintenance, and inspection fees. Precisely calculating these values is a difficult problem. Thus, in this work the authors selected values to reflect the anticipated ratio of costs for different errors, rather than precise cost values. One representative setting of these parameters is shown in Table 2.

As defined in Table 2, the database modification fee is set at $200. The remaining costs are defined differently for different cases. For critical situations, e.g., when a CS 3 (poor) is classified as CS 1 (good), the penalty is high while for the less consequential situations, e.g., where a CS 1 (good) is classified as CS 2 (fair), the penalty is low.

Once the costs are defined, the cost function can be used to calculate the total loss of utilizing this AI-based CS evaluation method:

(1) $$Cost=\sum_{i}\sum_{j}n_{ij}c_{ij}$$

In Equation (1), *i* (*i* = 1, 2, 3) indicates the true CS of the crack on the reinforced bridge deck, while *j* (*j* = 1, 2, 3) indicates the evaluated CS through the method proposed in this paper. $n_{ij}$ represents the number of misevaluations having a true label *i* but evaluated as *j*, while$\;c_{ij}$ represents the cost of this misevaluation and is defined in Table 2. 

### 2.4. Application of Deep Learning-Based Method as a Decision-Making Problem

In the previous section, a cost function was proposed to calculate the cost due to the limitations of this AI-based method. Since the primary attraction of implementing an AI method is to reduce the costs associated with a human’s work [39], it is important to develop a method to quantitatively analyze whether the inspector should trust any automated method. For every reinforced concrete bridge deck, an inspector must decide to either accept the automated result and its uncertainty, with the risk of overlooked degradation, or ignore the results in favor of manual inspection. The cost of manual inspection should be considered in this decision. Previous research considered how to make this decision for a very difficult problem involving data entry [39]. Thus, this method assumes that all classification steps prior to the semantic segmentation step are 100% accurate and all risk in this workflow stems from the semantic segmentation step. The ability of a stakeholder to accept risk is often categorized as unprepared, risk-neutral, risk-averse, or extremely risk-averse [40]. In this work, the extremely risk-averse category indicates that the inspector allocates the entire inspection budget for manual inspection. The risk-neutral category indicates that the inspectors do not require any extra compensation for taking on additional risk. The risk-averse category represents inspectors who like to pursue a balance between the costs and risks. The unprepared category indicates that the inspector accepts all risks associated with the AI-based method and uses it for all inspections. 

To analyze this problem, the impact of the semantic segmentation results was considered, which is the last step in the workflow shown in Figure 1. Thus, each image in this step is assumed to have some cracking. We assumed that in a given region, there were N reinforced concrete bridge decks requiring crack CS assessment and the indices $i\in \left\{1,\;2,\ldots ,N\right\}$ identify each bridge deck. The manual inspection fee for cracking on one bridge is denoted as k. The total cost of the CS assignment work can be determined using:

(2) $$C\left(x,\;r\right)=\sum_{i=1}^{N}\left[kz_{i}\left(r\right)+c_{i}\left(d_{i}\left(x\right),\;S_{i}\right)\left(1-z_{i}\left(r\right)\right)\right]$$

In Equation (2), x is the specific image of the bridge deck containing cracking. Here, we assumed that the condition state of the crack was only determined by one image. $d_{i}\left(x\right)$ is the CS predicted using image X, $c_{i}\left(d_{i}\left(x\right),\;S_{i}\right)$ is the cost corresponding to the *i*-th bridge deck when the predicted CS is $d_{i}$ and the true label is $S_{i}$, and $z_{i}\left(r\right)$ is a binary decision indicating whether to accept the predicted CS. The $z_{i}\left(r\right)$ value is determined by a pre-set manual check budget r, reflecting the risk preference of the engineer. For example, a risk-neutral inspector will want to minimize the total expected cost, which can be quantitatively represented by:

(3) $$\underset{r\in \left[0,\;\infty \right)}{\min}E\left[C\left(x,\;r\right)\right]$$

In Equation (3), $E\left[\cdot \right]$ indicates the expectation of the total cost over the random variables. Another example is an extremely risk-averse inspector, who may wish to keep the variance of the cost as low as possible. The variance is the indicator of the risk because the bridge deck inspection images obtained could be different every time. For example, even for the same bridge, changes in the background environment or weather conditions could occur over time. Though the approximate distribution of the images taken in each inspection period should be close, this difference, to some extent, will cause randomness in the results output from an AI-based method. However, this randomness does not influence the crack CS assessment result through the manual inspection herein as the manual inspection is assumed to be 100% accurate. Thus, increased participation for the AI-based method in crack CS assessment work produces larger randomness in the results, which is reflected by the variance of the total cost.

(4) $$\underset{r\in \left[0,\;\infty \right)}{\min}V\left[C\left(x,\;r\right)\right]$$

In Equation (4), $V\left[\cdot \right]$ indicates the variance of the total cost. The Pareto frontier, which is used in multi-objective optimization [41], is established to find the suitable pre-set manual inspection budget. To build the Pareto frontier, a list was built containing all budget levels, [$r_{0},\;r_{1},\ldots ,\;r_{N}$] and $r_{0}<r_{1}<\ldots <r_{N}$. Here, $r_{0}$ is 0, while $r_{N}$ refers to a budget large enough to inspect all *N* bridges in this region. Due to the randomness mentioned above, for each budget level we sampled *M* times to capture this randomness. The expected cost in Equation (3) can then be approximated by:

(5) $$E\left[C\left(x,\;r\right)|r=r_{n}\right]\approx \bar{C}\left(r_{n}\right)≔\frac{1}{M}\sum_{m=1}^{M}C\left(x,\;r=r_{n}\right)$$

The variance in Equation (4) can be calculated by:

(6) $$V\left[C\left(x,\;r\right)|r=r_{n}\right]\approx \sigma_{C}^{2}\left(r_{n}\right)≔\frac{1}{M}\sum_{m=1}^{M}{\left\{C\left(x,\;r=r_{n}\right)-\bar{C}\left(r_{n}\right)\right\}}^{2}\;$$

With the equations for the expected cost and the variance of the cost for each budget level, the Pareto frontier can be plotted to visualize the relationship among the risks (variance), the total costs (expected costs), and the pre-set manual inspection budget. The bridge inspector can then use this plot with the Pareto frontier to determine the budget for the element-level CS assignment for reinforced concrete deck cracking.

## 3. Experimental Validation

### 3.1. Description of the Dataset

To train and validate each step in the method, a ground truth dataset composed of 3549 images was manually labelled. This dataset included 2350 “Cracked” images and 1199 “non-Cracked” images. The 2350 images in the “Cracked” category were further categorized and labelled into 1660 “non-Sealed” images and 690 “Sealed” images. Furthermore, the 1660 “non-Sealed” were categorized and labelled into 798 “Normal Crack” images and 862 “Map Crack” images. Recall that all “non-Sealed” images were also labelled for semantic segmentation for the crack contours. These images were annotated using tools in Labelbox [42]. All images in this dataset were captured during real bridge inspections and collected from the BIAS [33] established by the INDOT.

### 3.2. Settings of Each Step in the Workflow

#### 3.2.1. Image Classifiers in the First Three Steps

As described in the section “Design of the AI-based Method”, image classification is used in the first three steps of the workflow. For this application, ResNet50 [43] in Keras [44] was selected as the baseline model for training the classifiers considering the performance of the model and the capacity of our hardware, though there are several reasonable models that may be adopted. ResNet50 is a deep residual neural network architecture consisting of 50 layers and employing residual connections, which allow for the creation of very deep neural networks while preventing the problem of vanishing gradients. The architecture consists of a stack of convolutional layers followed by batch normalization, ReLU activation, and max pooling layers. The core of the network is composed of residual blocks, which contain two or three convolutional layers with batch normalization and ReLU activation, along with skip connections that bypass the convolutional layers. Before training, the images for each classifier were split into a training set and a validation set with the ratio of 4:1. Five-fold cross validation was implemented to eliminate bias caused by dataset split. A NVIDIA GPU GeForce GTX Titan X was utilized to perform the training.

#### 3.2.2. Semantic Segmentation in the Last Step

In the final step of the workflow, semantic segmentation was used to detect the crack contours of “Normal Crack” and “Map Crack” images. For this method, images are typically downsized due to GPU memory limitations. When looking for cracks that are very small, however, resizing the image will blur or entirely erase fine crack details as they may only be several pixels in width. To avoid this problem, researchers have had success by cutting a single image into a batch of smaller images that can be used with machine learning algorithms [45]. This technique decreases the pixel number for each image being processed without losing any pixel information. For this implementation, the original images were cut into smaller batch images of 64 × 64 pixel dimensions. 

As the cracks usually only take up a small part of the original image, a large portion of the 64 × 64 pixel batch images may not show the cracks. To remedy this, a fourth image classifier distinguishes whether the batch image contains cracking or not. To build this classifier, 206 images in the “Cracked” category of our dataset were randomly selected to build the training and validation set. The cracks were annotated in these images in the first step and the images were then cropped into batch images. Batch images containing more than 400 crack pixels were labeled as “Crack Batch” images and other batch images were labeled as “Non-Crack Batch”. Since the number of “non-Crack Batch” images was far greater than the number of “Crack Batch” images after cropping, to balance the dataset around 15,000 “non-Crack Batch” images were randomly selected to train with 8713 total “Crack Batch” images. Considering the smaller size of batch images, a more complex baseline model was selected to leverage the capacity of the GPU and improve the performance of the classification. Thus, the network EfficientNetB7 [46] was used as the baseline model to train the classifier for “Crack Batch” and “non-Crack Batch” images. EfficientNetB7 is the largest model in the EfficientNet family, with a total of 66 million parameters. It used a compound scaling method that balanced the depth, width, and resolution of the network to improve its overall efficiency. EfficientNetB7 also consisted of a series of stacked blocks, each containing a combination of convolutional layers, batch normalization, and activation functions.

**Figure 5:**
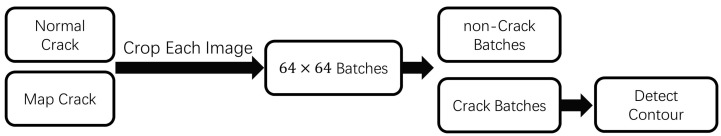
Detailed process for semantic segmentation in final step.

Once a batch image was determined to show a crack, the segmentation process could proceed. The MaskRCNN [47] network was used as the baseline model, considering the performance of the model and the capacity of our hardware, to train for detecting the contour of the cracks in each batch image. Mask R-CNN is a two-stage deep learning architecture. The first stage serves as a region proposal network (RPN) that generates candidate object proposals, while the second stage served as a refinement network that performs object detection and segmentation on these proposals. The refinement network was composed of a series of convolutional layers, which extracted features from the input image, followed by a set of regions of interest (ROI) pooling layers, which extracted features from each proposed object region. In addition, Mask R-CNN added a parallel branch to the refinement network that predicts the segmentation mask for each detected object. This branch was composed of a set of convolutional layers that refine the feature maps generated by the ROI pooling layers, followed by a fully connected layer that outputs a binary mask for each object region proposal. As for the Image Classifiers I, II, and III, the dataset was split into a training set and a validation set with the ratio of 4:1. Five-fold cross validation was implemented to eliminate the bias caused by dataset split. A Nvidia GPU GeForce GTX TITAN X was also utilized to conduct the training work in this last step of the workflow. The overall process used in this final step is shown in Figure 5:

### 3.3. Results

#### 3.3.1. Result for Step 1

The first step used Image Classifier I to determine if the bridge deck shown in an image contained a crack or not. The average classification accuracy among five different dataset splits was approximately 0.94. The average precision and recall among five different dataset splits were 0.93 and 0.94, respectively. One example of the classification results for Step 1 is shown in the confusion matrix in Table 3:

#### 3.3.2. Result for Step 2

The second step used Image Classifier II to determine if the crack detected had been sealed or not. The average classification accuracy among five different dataset splits obtained for this classifier was approximately 0.93. The average recall and precision among five different dataset splits were around 0.92 and 0.91, respectively. One example of the classification results for step 2 is shown in the confusion matrix in Table 4:

#### 3.3.3. Result for Step 3

The third step used Image Classifier III to determine if an unsealed crack on the concrete bridge deck is a “Normal Crack” or “Map Crack”. The average accuracy for this step was approximately 0.84 among five different dataset splits. The average recall and precision among five different dataset splits were 0.85 and 0.84, respectively. One example of the classification results for step 3 is shown in the confusion matrix in Table 5:

#### 3.3.4. Result for Step 4

**Figure 6:**
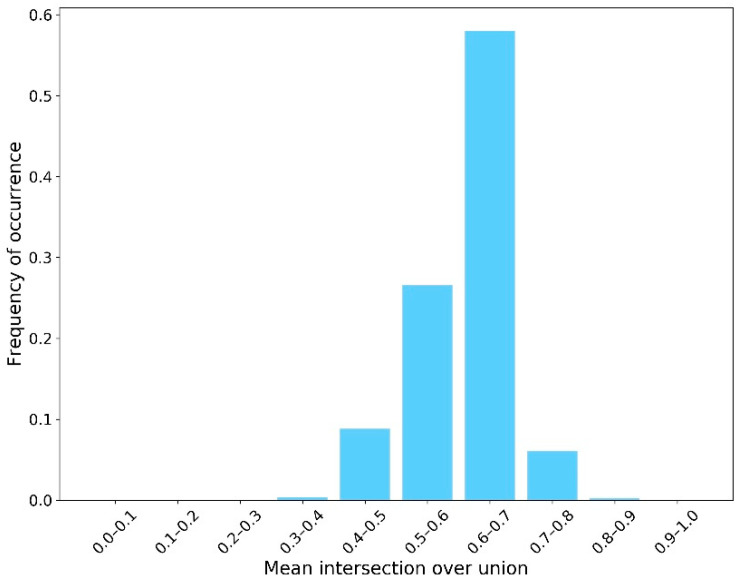
Distribution of mean IoU.

**Figure 7:**
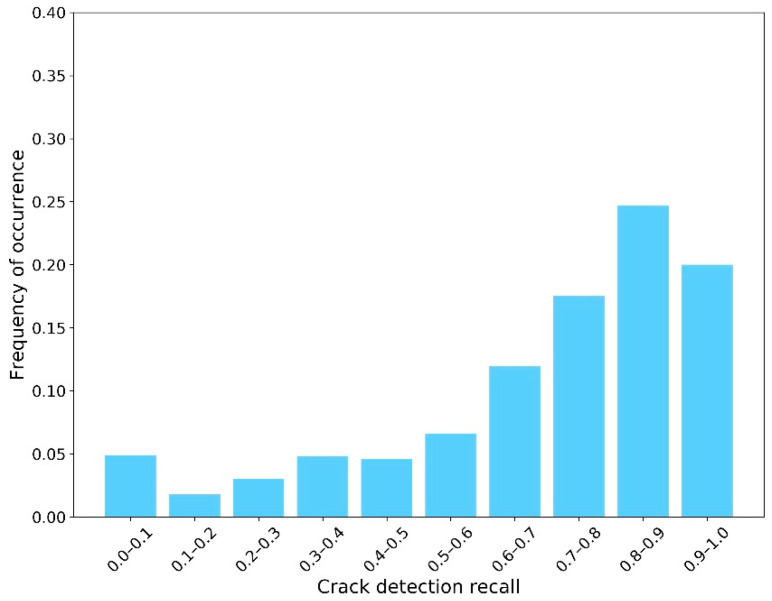
Distribution of recall.

**Figure 8:**
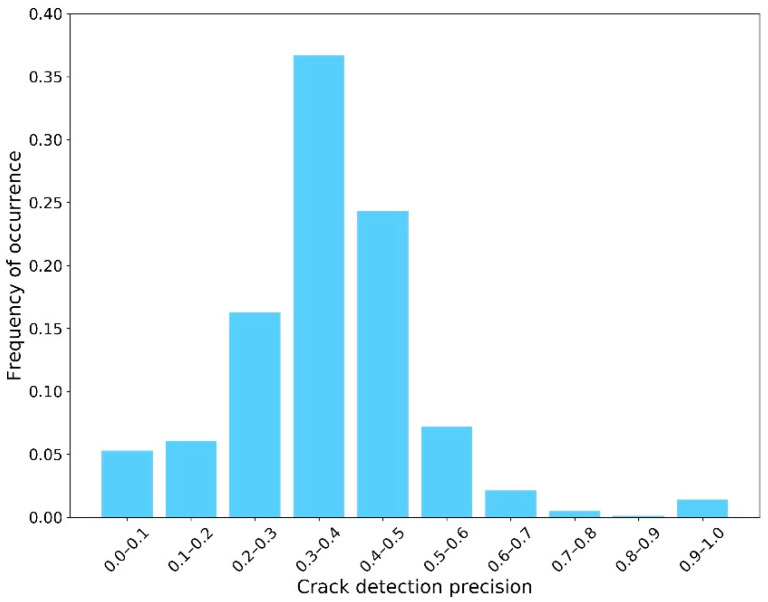
Distribution of precision.

As demonstrated previously, the final step in the workflow involved two sub-steps. The first sub-step used another image classifier to determine if a batch image was “Crack Batch” or “non-Crack Batch”. The average classification accuracy among five different dataset splits achieved was approximately 0.90. The average recall and precision were both 0.89. One of the classification results for this sub-step is shown in the confusion matrix in Table 6:

The second sub-step involved implementing the semantic segmentation algorithm on the “Crack Batch” images to detect the crack contours. For this, the average IoU was approximately 0.61. The average crack pixel detection recall and precision were 0.68 and 0.35, respectively. These semantic segmentation results were not as high as those of similar research. However, as mentioned in the section “Design of AI-based Workflow”, achieving high-performance models was not the goal of this paper since the images used for training were not specifically taken for crack inspection. Instead, these imperfect results were used in this work to exhibit their influence on the CS decision making process. Figure 6, Figure 7 and Figure 8 show the distribution of IoU, recall, and precision, respectively.

### 3.4. Improving Crack Type Classification

As shown in the “Results” section, the average accuracy of Image Classifier III, which distinguishes normal cracking and map cracking, was 0.83. This lower accuracy was likely a result of resizing the images before they were input to the convolutional neural network. As described previously, cracks may be quite thin and, thus, resizing the image can cause feature loss or even the disappearance of a crack, which could influence the classification results. To improve the results, images that showed only a small portion of cracking may be filtered out as these images are most affected by the re-sizing process.

To conduct the filtering work, the results of the classifier used in the final segmentation step, which distinguished small batch images as “Crack Batch” versus “Non-crack Batch”, were used to calculate an $R_{c}$ using Equation (7). This value represents an estimate of the percentage of “Crack Batch” in an image, rather than the percentage of exact cracking area.

(7) $$R_{c}=\frac{Number\;of\;Crack\;Batch\;Images}{Total\;Number\;of\;Batch\;Images\;in\;One\;Image}$$

**Figure 9:**
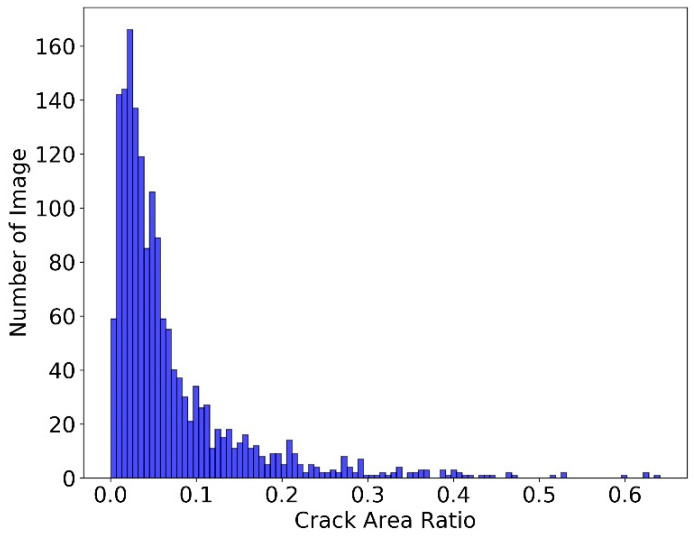
Distribution of $R_{c}$ in dataset of step 3.

If $R_{c}$ is small, the “Crack Batch” images make up only a small part of the whole image, which is not deemed suitable for this machine learning-based analysis using resizing. The distribution of $R_{c}$ in the dataset of Step 3 is shown in Figure 9.

As shown in Figure 9, $R_{c}$ ranged from 0 to 0.65. However, the number of images decreased rapidly after achieving the peak. Thus, if $R_{c}$ was too large, these images were deemed as “not suitable for machine learning-based analysis” because there were very few similar images in the dataset. An upper and lower threshold were applied to filter out the images unsuitable for machine learning-based analysis. The lower threshold and the upper threshold were set as 0.01 and 0.35, respectively. Images with $R_{c}$ < 0.01 or $R_{c}$ > 0.35 were then excluded from the machine learning training and validation process. Those excluded images could be manually inspected by bridge inspectors to determine their classifications. Different settings of the lower and the upper thresholds were examined in this study to identify an appropriate choice for this application.

As shown in Table 7, the classification accuracy was improved using this method. However, the accuracy stopped increasing when the range of thresholds was smaller than $0.01<=R_{c}<=0.35$. One reason for this saturation may be that as the range of thresholds narrowed, the impact of $R_{c}\;$became lower. Another explantation may be that the number of images used for training and validation became smaller while the range narrowed, which could influence the model performance. Due to limitations in the size of the dataset in this work, the primary reason for this could not be determined. However, the result still proved that with guidance based on the $R_{c}$, inspectors could properly participate in the crack type classification process and improve the performance of the machine learning model.

## 4. Illustrative Example

The previous section illustrated the results of each step in the proposed methodology. In this section, the methodology is used to demonstrate its application in practice. Furthermore, this section demonstrates how inspectors can plan their work based on their risk and cost requirements. As outlined in the “Technical Approach” section, the Pareto frontier is generated to assist the inspector in this decision making process. To produce the Pareto frontier, a manual inspection fee k of $1000 was assumed, which included labor, transportation, and equipment. The cost table defined in Table 2 is used to quantify the cost of misclassifying CS.

### 4.1. Establish an Synthetic Inventory

For this demonstration, a synthetic inventory of “images” was created. Recall that, for the decision making process, this paper focuses on the last semantic segmentation step. Thus, only crack width influences the CS results. The sample inventory was assumed to include non-sealed crack “images” with a variety of crack widths as only non-sealed crack images are input into the last step. Please note that these “images” are not real data but image pseudo-data used for simulation. This synthetic inventory includes three different categories of crack widths: insignificant (width < 0.012 inch), moderate (0.012 inch ≤ width < 0.05 inch), and wide (width ≥ 0.05 inch). The inventory was established with a uniform crack-width distribution, as shown in Table 8, using one-thousandth of an inch as the smallest increment. Each possible crack width was correlated to 20 images. One crack image was assumed to be correlated with one bridge in the inventory. The resulting inventory contains 1235 “bridges”, with the number of CS1, CS2, and CS3 crack images being 220, 760, and 255, respectively.

### 4.2. AI-Based Crack Width Detection Simulation Process

After establishing the inventory, the quantitative results from the “Experimental Validation” section were utilized to simulate the AI-based crack width detection process. When calculating the performance score of a semantic segmentation model, the pixel-level confusion matrix illustrated in Table 9 was used as an intermediate value to record the true pixel label and predicted pixel label. 

In this table, BG Pixels are background pixels referred to as non-crack pixels of an image. TP is True Positive, FN is False Negative, FP is False Positive, and TN is True Negative. The recall and precision of crack prediction can be calculated through the following two equations:

(8) $$Recall_{crack}=\frac{TP}{TP+FN}$$

(9) $$Precision_{crack}=\frac{TP}{TP+FP}$$

Through the pixel-level confusion matrix and the equations of recall and precision, the number of true crack pixels (defined as TP + FN) were calculated from predicted crack pixels (defined as TP + FP) using:

(10) $$Number\;of\;True\;Crack\;Pixels=\frac{Number\;of\;Predicted\;Crack\;Pixels\times Precision_{crack}}{Recall_{crack}}$$

The above equation can be derived to:

(11) $$Number\;of\;Predicted\;Crack\;Pixels=\frac{Number\;of\;True\;Crack\;Pixels\times Recall_{crack}}{Precision_{crack}}$$

The difference between the predicted crack pixels and the true crack pixels could happen in the direction parallel to the crack (lengthwise) or perpendicular to the crack (widthwise). For the CS assignment work in this paper, only the prediction errors in the crack-width direction have an impact because the assignment of CS only requires the width of a crack, per *MBEI*. Thus, to simulate the worst-case scenario the prediction errors were assumed to happen only in the widthwise direction, while the prediction of crack lengths were assumed to be 100% accurate. Equation (11) was then developed into:

(12) $$Prediction\;Crack\;Width=\frac{True\;Crack\;Width\times Recall_{crack}}{Precision_{crack}}$$

The $Recall_{crack}$ and $Precision_{crack}$ are randomly picked from the results obtained in our experimental validation step. The simulation of the AI-based crack width detection process was then conducted using the pseudo code shown in Algorithm 1:
**Algorithm 1:** Simulation for AI-based Crack Width Detection Process**Input:** Semantic Segmentation Model Recall and Precision Distribution; Synthetic Inventory**Output:** Predicted Crack Width 1: for crack_image **in** inventory **do**: 2:   True crack width 🡰 crack_image 3:   Recall 🡰 randomly pick from semantic segmentation model recall distribution 4:   Precision 🡰 randomly pick from semantic segmentation model precision distribution 5:   Predicted crack width 🡰 $\frac{True\;crack\;width\;*\;Recall}{Precision}$

### 4.3. CS Assignment Simulation Process and Results

In this approach, once the predicted crack width is obtained, the predicted CS of the crack is assigned to the crack image and, thus, to the bridge. The predicted CS is then compared to the true CS and the cost of the prediction is taken from cost table defined in the section “Cost Function to Quantify Loss Due to Misclassification of Condition States”. By pre-setting a manual inspection budget, which is denoted as r, some crack images in the inventory undergo manual inspection, while the rest of images are examined by the AI-based method. In this example, as the manual inspection fee for one bridge was defined as $1000, the budget level ranged from $0 for an unprepared inspector to $1,235,000 for a fully risk-averse inspector doing only manual inspection. The value of manual inspection fee is only selected as an example to implement the simulation process and, in practice, this value should be determined by local economic level. For each budget level, the crack width detection process was run 1000 times to capture the randomness existing in the AI-based method. The cost expectation and the cost variance under each budget were then calculated. Algorithm 2 represents the pseudo-code of the CS assignment simulation process under one budget level.
**Algorithm 2:** Simulation for CS Assignment Simulation Process**Input:** Budget, r; **Output:** Cost Expectation, E; Cost Variance, V1: **for** $i\in \left[1,\;2,\;3,\ldots ,\;1000\right]$ **do**: 2:   All Predicted Crack Widths 🡰 Simulation for AI-based Crack Width Detection Process 3:   Manually Inspect Bridges 🡰 Randomly Select based on r and the Prediction Results 4:   $Cost_{i}$ 🡰 r + Misassignment Cost of CS for Rest of Bridges 5: E 🡰 $\sum_{i=1}^{i=1000}Cost_{i}$/1000 6: V 🡰 $\sum_{i=1}^{i=1000}{\left(Cost_{i}-E\right)}^{2}$/1000

**Figure 10:**
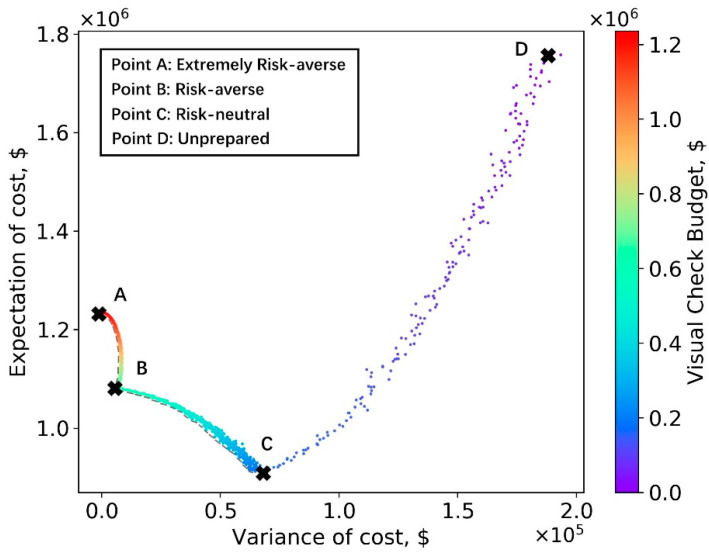
Simulation results: cost expectation vs. cost variance.

We note that, in practice, the bridge inspector especially wants to avoid more critical and nonconservative errors, e.g., when a CS3 (poor) is assigned as a CS1 (good). It follows that bridges that are predicted as CS1 should be the first ones to be manually inspected. If the pre-set budget is sufficient to check all CS1 images, the inspector can move onto manually inspecting those predicted as CS2, etc. As described in the “Application of Deep Learning-based Method as a Decision-making Problem” section, the Pareto frontier was established with the cost expectation and cost variance obtained via the above mentioned simulation process. The results of CS assignment simulation under each budget level are shown in Figure 10.

Bridge inspectors can use this figure to adjust the manual inspection budget and tailor the results to their risk preference. The risk (indicated by variance of cost) at point A is zero and, thus, the extremely risk-averse inspectors should adopt the budget at point A, which is $1,235,000. On the opposite end of the spectrum, point D does not prepare any manual inspection budget and all bridge decks are checked using the AI-based method, indicating unprepared inspectors. Obviously, points A and D do not minimize the total cost, which is the aim of the risk-neutral inspector. Instead, the budget set in point C corresponds to this goal and, in this case, the value of this budget is around $200,000. The risk-averse inspector prefers to minimize both the expectation and the variance of the total cost. To achieve this goal, the Pareto frontier [41] is represented by the dashed line. Based on the results shown, the authors recommend that the risk-averse inspector sets the manual budget at $600,000. However, the risk-averse inspector might select any budget located on the efficient frontier.

### 4.4. CS Discussion of Results

**Figure 11:**
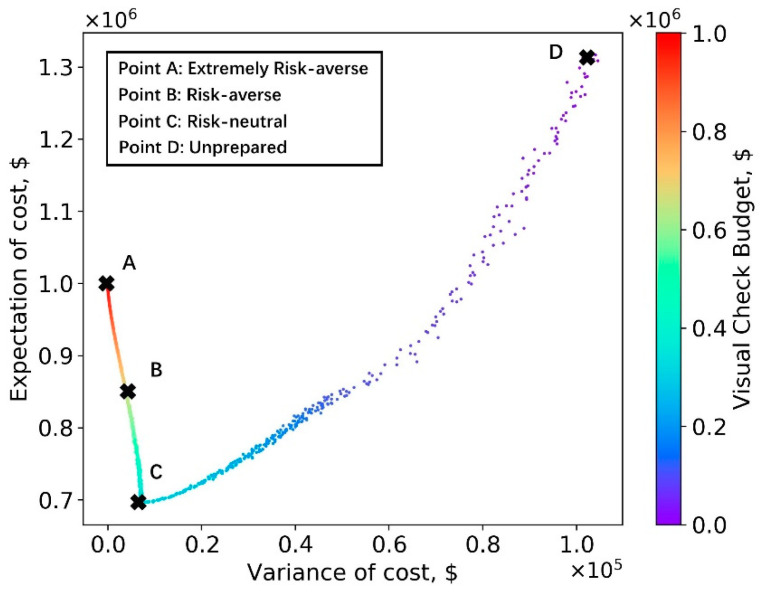
Expectation vs. variance with different datasets and ratios among different misassignment scenarios and inspection fee.

The expectation and variance of the total cost shown in Figure 10 is the result of many factors, including the distribution of the crack CS in the synthetic dataset, the relative cost ratios for misassignment scenarios, and the inspection fee (defined in the cost table). To demonstrate this point, the CS assignment simulation defined in Algorithms 1 and 2 was run for an alternate synthetic inventory, which contains 110 CS1 bridges, 380 CS2 bridges, and 510 CS3 bridges; this simulation represents a scenario where a larger fraction of the decks is in poor condition. A distinct cost table has been chosen to account for the anticipated ratio of costs for several errors in an alternate region. This cost table is shown in Table 10, while the simulation results are shown in Figure 11.

As shown in Figure 11, with different settings for the synthetic dataset, cost of misassignment, and inspection fee, the inspector can still use their resources more efficiently on the total costs using the AI–based method. However, the appropriate budgets for extremely risk-averse, risk-averse, and risk-neutral categories are not the same as the results shown in Figure 10. Other settings were also tried during this simulation experiment and they produce a variety of expectation and variance plots. For example, if the misassignment cost is large, choosing human inspection is more desirable. Thus, different bridge inspectors should set their budgets while fully considering their own inventory, risk preferences, and relative costs. 

**Figure 12:**
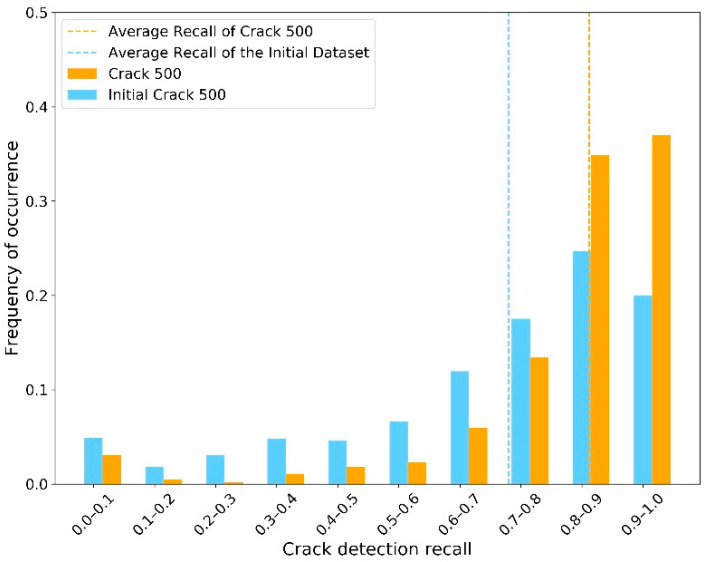
Distribution of recall with dataset Crack 500, compared to initial dataset.

**Figure 13:**
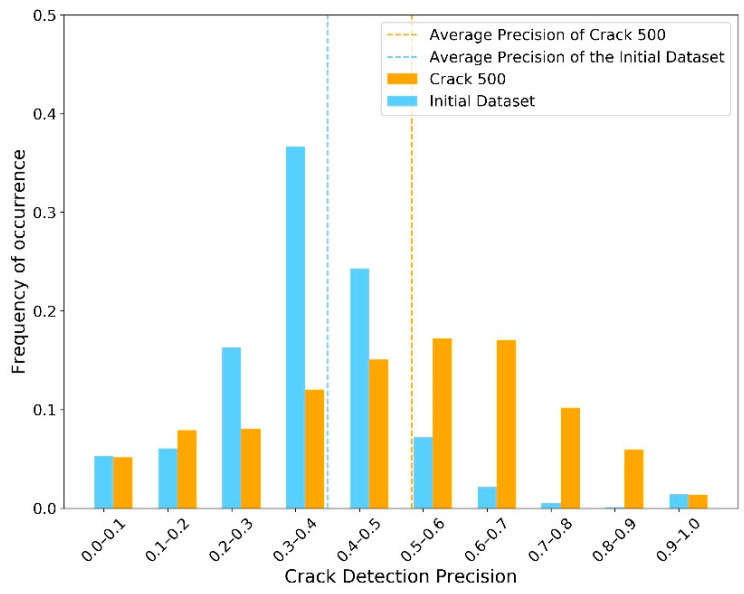
Distribution of precision with dataset Crack 500, compared to initial dataset.

Another important point to consider is that these plots were created using relatively inaccurate semantic segmentation results, when compared with the existing body of research, due to the nature of the real-world inspection images used. These images were not taken for the purpose of determining the condition state of cracks and contain a lot of miscellaneous information and noise. To illustrate this point and its impact on the simulation process, the authors implemented the same training process on another publicly available dataset—Crack 500 [48]—which was specifically built for concrete crack inspection (not just for concrete bridge decks). The average IoU, recall, and precision increased to 0.70, 0.82, and 0.48, respectively. As shown in Figure 12 and Figure 13, the distribution of recall and precision was significantly improved. 

**Figure 14:**
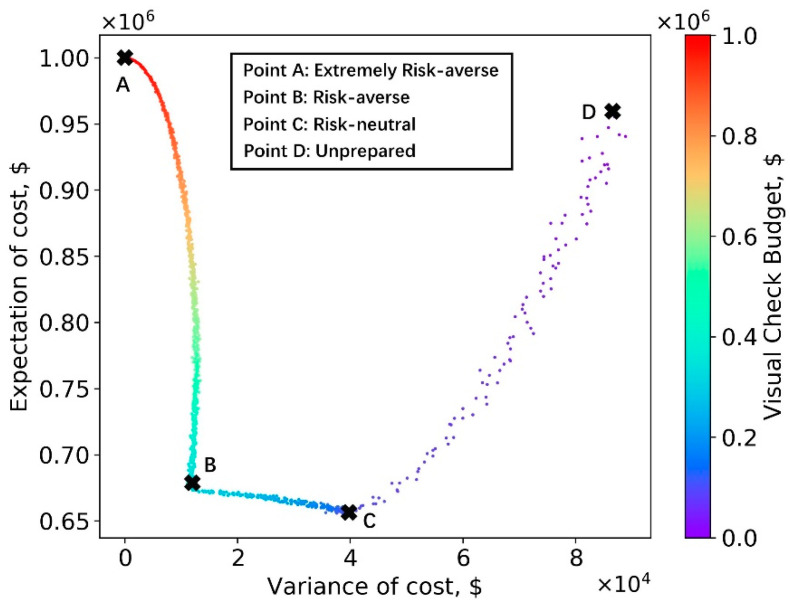
Expectation vs. variance with a better semantic segmentation model.

The outcome of using a better performance distribution in the simulation process is shown in Figure 14. This simulation used the same dataset and cost table used in Figure 11. Clearly, Figure 14 indicates that it is acceptable to completely replace manual participation with the AI-based method. The total cost in point D is lower than point A. Thus, it is suggested that inspectors take clear crack images during the inspection process, considering the potential economic benefits of eventually implementing a mostly AI-based method.

The examples provided in this paper only serve as references. Bridge asset inspectors who plan to use the method developed here should adjust the cost table based on their own regional costs and inventory. Overall, these results show that there are advantages to having the inspectors and machines work together for CS assignment tasks.

## 5. Conclusions

With the requirements of the newly released *SNBI 2022*, bridge inspection involves more tasks and these tasks require more resources. Thus, an AI-based workflow was developed and validated to assist inspectors with one of these new tasks: the condition state assignment of a concrete bridge deck cracking. The workflow utilizes three cascading levels of CNN–based binary classifiers to distinguish cracked deck from non-cracked deck images (step 1), sealed crack from non–Sealed crack images (step 2), and normal cracking from map cracking images (step 3). Semantic segmentation is then used to generate the contour unsealed crack images (step 4). Based on the results, each image is assigned a condition state in accordance with the *SNBI 2022*. Classification accuracies of 0.94, 0.93, and 0.84 were achieved for a dataset of real-world inspection images for steps 1, 2, and 3, respectively, validating these steps of the workflow. The final step yields a calculated IoU of 0.61. This outcome occurred because the images used were not taken for the purpose of applying machine vision and studying cracks, as shown by their much better performance when the methods were used on the Crack 500 dataset that was designed for this purpose. Rather, the purpose of this step was not to optimize the results but to illustrate the overall workflow and the impact of the results on its application when realistic images collected in the field are used.

Inspectors are unlikely to choose full automation, especially in the case of high-risk bridge inspections. Thus, in addition to the workflow, a decision making method was developed which enables bridge inspectors to adjust the inspection budget and their risk preferences to strike the right balance between automated and manual inspection. This tool was used in an illustrative example where we also varied the cost settings and the quality of the semantic segmentation results as discussed above. The influence of these factors on the costs and risks of applying the AI-based workflow was analyzed and discussed.

Based on this study, the authors have several implementation recommendations for bridge inspectors adopting this method. Firstly, high-quality crack images may be taken by inspectors to optimize the performance of the workflow. While the illustrative example showed that even AI models with low performance can help in inspection work, enabling higher performance by taking higher quality crack images will certainly further reduce the costs and risks. Secondly, pre-analysis of the image before applying this workflow is a necessary step to improve overall performance. An inspector should view all outliers and remove those images not suitable for the workflow, while the AI models can still deal with the rest of the massive data. In this way, an effective collaboration is established that can take advantage of both humans and AI to optimize performance. Thirdly, to apply the method in practice different DOTs should collect information on their inspection costs and evaluate their risk tolerance so that they can make use of the method for their needs. Overall, this work illustrated the practical application of a framework for evaluating concrete bridge deck cracking. 

Finally, in this work the CS of the crack is only determined by one image whereas, in reality, bridge inspectors may take several images for the same area. Thus, a method for fusing information from multiple images can be developed in the future, which would likely improve the robustness of the method. Additionally, the costs were analyzed based on the assumption that the classification steps were perfect. In reality, although it is a well-established technique, the accuracy of classification steps will also influence the decision of budget setting. Thus, people can also include such influence in the future. However, the basic idea of the decision making process for practical problems is the focus of this paper. Researchers may also expand these functions for other types of damage and other bridge elements.

## Figures and Tables

**Table 1:** Cost table for quantifying classification of CS for deck cracking.

		Predicted CS		
		CS 1	CS 2	CS 3
**True CS**	**CS 1**	$C_{MIS11}$	$C_{MIS12}+C_{modify}$	$C_{MIS13}+C_{modify}$
	**CS 2**	$C_{MIS21}+C_{modify}$	$C_{MIS22}$	$C_{MIS23}+C_{modify}$
	**CS 3**	$C_{MIS31}+C_{modify}$	$C_{MIS32}+C_{modify}$	$C_{MIS33}$

**Table 2:** A representative cost table with specific values.

		Predicted CS		
		CS 1	CS 2	CS 3
**True CS**	**CS 1**	$0	$1000+$200	$1500+$200
	**CS 2**	$25,000+$200	$0	$1000+$200
	**CS 3**	$50,000+$200	$25,000+$200	$0

**Table 3:** Confusion matrix for step 1 classification.

Confusion Matrix		Predicted Label	
		Cracked	Non-Cracked
**True Label**	**Cracked**	449	24
	**Non-Cracked**	17	220

**Table 4:** Confusion matrix for step 2 classification.

Confusion Matrix		Predicted Label	
		Non-Sealed	Sealed
**True Label**	**Non-Sealed**	314	15
	**Sealed**	17	124

**Table 5:** Confusion matrix for step 3 classification.

Confusion Matrix		Predicted Label	
		Normal Crack	Map Crack
**True Label**	**Normal Crack**	194	61
	**Map Crack**	20	221

**Table 6:** Confusion matrix for step 4 classification.

Confusion Matrix		Predicted Label	
		Crack Batch	Non-Crack Batch
**True Label**	**Crack Batch**	1484	219
	**non-Crack Batch**	264	2902

**Table 7:** Model performances with different settings of thresholds.

$\mathrm{Threshold}\;\mathrm{of}\;R_{c}$	Computer Classification Accuracy	Number of Images Exempted
$0<=R_{c}<=1$	0.840	0 out of 1660
$0.01<=R_{c}<=1$	0.872	135 out of 1660
$0.01<=R_{c}<=0.35$	0.896	167 out of 1660
$0.01<=R_{c}<=0.30$	0.882	181 out of 1660
$0.02<=R_{c}<=0.35$	0.887	391 out of 1660
$0.02<=R_{c}<=0.3$0	0.890	405 out of 1660

**Table 8:** Synthetic inventory for building Pareto frontier.

Bridge Id	Crack Width	Condition State
1–20	0.001 in	1
21–40	0.002 in	1
41–60	0.003 in	1
…	…	…
201–220	0.011 in	1
241–260	0.012 in	2
261–280	0.013 in	2
281–300	0.014 in	2
…	…	…
961–980	0.049 in	2
981–985	0.05 in	3
986–990	0.051 in	3
…	…	…
1231–1235	0.1 in	3

**Table 9:** Pixel-level confusion matrix.

Pixel—Level Confusion Matrix		Predicted Label	
		Crack Pixels	BG Pixels
**True Label**	**Crack Pixels**	TP	FN
	**BG Pixels**	FP	TN

**Table 10:** Cost table for a different scenario.

		Predicted CS		
		**CS 1**	CS 2	CS 3
**True CS**	**CS 1**	$0	$1500+$200	$1500+$200
	**CS 2**	$10,000+$200	$0	$1500+$200
	**CS 3**	$20,000+$200	$10,000+$200	$0

## Data Availability

Restrictions apply to the availability of these data. Data was obtained from Indiana Department of Transportation (INDOT) and are available at: https://indot-it.bentley.com/login.aspx (accessed on 1 January 2023) with the permission of INDOT.
